# Characterization of the Key Aroma Constituents in Fried Tilapia through the Sensorics Concept

**DOI:** 10.3390/foods11040494

**Published:** 2022-02-09

**Authors:** Mingyuan Liu, Xiaoying Zhao, Mouming Zhao, Xiaoling Liu, Yiyang Pang, Meishuo Zhang

**Affiliations:** 1 Department of Food Science, Guangxi University, No. 100, Daxue Road, Nanning 530004, China; jacoblmy@163.com (M.L.); zxy16896@163.com (X.Z.); zmmgxu@gxu.edu.cn (M.Z.); pyyangur@163.com (Y.P.); zms0206@foxmail.com (M.Z.); 2 College of Light Industry and Food Sciences, South China University of Technology, No. 381 Wushan Road, Guangzhou 510640, China

**Keywords:** fried tilapia, aroma extract dilution analysis, accelerated solvent extraction, high vacuum flavor extraction technology, odor-active compounds

## Abstract

The object of this study was tilapia fish that were fried in soybean oil. Volatile compounds were extracted from the fish by ASE-HVE and were studied by GC-O-MS and the AEDA analysis method. A total of 30 aroma compounds were initially determined, and these compounds contribute to the aroma of fried tilapias. The key volatile compounds in fried tilapia were quantitatively analyzed by GC-MS, and the volatile compounds in soybean-fried tilapia were studied by flavor recombination and deletion experiments. Trimethylamine, hexanal, 2,3-dimethylpyrazine, dimethyl trisulfide, trans-2-octenal, 2,3-dimethyl-5-ethylpyrazine, (E)-2-nonenal, 2-propyl-pyridine, and (E,E)-2,4-decadienal were finally determined to be the key volatile compounds in soybean-fried tilapia.

## 1. Introduction

*Nile tilapia* (*Oreochromis niloticus*) is the second highest economically farmed fish because it has good adaptability and can be raised in harsh conditions [1]. In 2016, Guangxi produced 308,000 tons of tilapia, accounting for 17.3% of China’s total output; 25 to 30% of this output was exported to developed countries, such as Europe and the United States (USA), and the rest was used for local or regional consumption [2].

The crispiness of the fried product, the aroma of baking and frying, and the pleasant golden to brown color make fried tilapia a popular food worldwide [3]. The smell of food is an important index in food evaluation, so the study of fried food is very important [4]. Scientists around the world have carried out much research on the characteristics of aquatic products in regard to their odors. However, there are few studies on volatile flavor compounds in tilapia or other aquatic products during thermal processing. Salum et al. explored raw and cooked red mullet through combined direct solvent extraction and solvent-assisted flavor extraction [4]. Mall et al. explored the key aroma compounds in raw and thermally processed prawns and thermally processed lobsters by utilizing aroma extract elution analysis [5]. Lapsongphon et al. explored the aroma-impact components of Thai fish sauce [6]. Cayhan and Selli explored the key aroma compounds in cooked *grey mullet* by applying aroma extract dilution analysis [7]. However, these studies aimed to discover the essential aspects of the quantitative analysis of volatile compounds, and missing restructuring and experimental model are nonexistent; thus, the key components cannot be validated as volatile compounds. There is a lack of convincing evidence that could provide guidance for industrial quality control. The key products in fried aquatics have not been extensively researched regarding volatile compounds, and the key volatile compounds of fried aquatic products have not been studied in depth.

The specification of aromatic volatile compounds may have differences based on the extraction technique utilized. Many flavor substance extraction methods have been developed [8,9,10]. High empty-card solvent-assisted flavor evaporation devices have a good smell contour reduction effect and low extraction temperature and can effectively reduce the enzyme-containing raw materials while generating interference in the extraction process. Many flavor compounds have been applied in the study [11,12,13,14,15]. However, a high overall cost and high cost of maintenance are necessary for utilizing this device, leading to substantial resistance of its application.

In this study, after tilapia were fried in soybean oil, their muscles were taken as the research subject, focusing on the following: (1) the extraction of flavor substances was carried out by accelerated solvent extraction and high-vacuum flavor extraction technology (ASE-HVE), the HVE device independently manufactured by our laboratory; (2) the research methods of GC-MS-O and aroma extract dilution analysis (AEDA) were used to explore the key volatile compounds in fried tilapia; (3) a quantitative analysis of volatile compounds in deep-fried tilapia was conducted by GC-MS; (4) OAV values were used to validate key volatile flavor compounds; (5) GC-O and GC-MS analysis results were verified by aroma recombination tests; and (6) omit studies were conducted to determine which odorant-active compounds had the greatest effect on the overall aroma.

## 2. Materials and Methods

### 2.1. Chemical Standards

Methanol (p.a.) and sodium sulfate (p.a.) were purchased from Sinopharm Chemical Reagent Co., Ltd. (Shanghai, China). The following reference compounds were purchased from Merck Group (Shanghai, China): dichloromethane (99.8%), N,N-dimethylmethanamine (p.a.), and chloroform (p.a.) 2-methyl-1-propanal, diacetyl, hexanal, 2-pentyl furan, amyl alcohol, 2-methyl pyrazine, octanal, acetone alcohol, 2,6-dimethyl pyrazine, 2-ethyl pyrazine, 2,3-dimethyl pyrazine, dimethy trisulfide, nonanal, 2-ethyl-6-methyl pyrazine, 2,3,5-trimethyl pyrazine, trans-2-octenal, 1-octen-3-one, 2,3-dimethyl-5-ethylpyrazine, 2,3,5,6-tetramethyl pyrazine, benzaldehyde, (E)-2-nonenal, 2-propyl-pyridine, gamma-butyrolactone, furan-2-ylmethanol, 2-hexylthiophene, 2-undecenal, (E,E)-2,4-decadienal, and (R)-(-)-pantolactone. The alkane mixes C5-C40 were purchased from Anpel (Shanghai, China). The purity of these and the aforementioned compounds was not less than 97%.

### 2.2. Preparation of Fried Tilapia

The body weight of tilapia was approximately 1.5 kg (the tilapia was identified by Professor Liu of Guangxi University). After slaughtering the tilapia, the skin, skeleton, heads, and meat were removed. The tilapia was put into a HP-12 meat grinder (Wenzhou, China) and ground into minced meat.

The minced meat was stored in refrigerator at 4 °C. Twenty grams of tilapia minced meat was kneaded into 2-cm diameter meatballs. The treated tilapia was added to soybean oil (COFCO, Shanghai, China) that was heated to 180 °C in advance and fried for 7 min. Then, the tilapia was removed and drained for 5 min. Dried tilapia minced meat was then added to soybean oil that was heated to 140 °C, and the meat was fried for 2.5 min. Then, the meat was removed and drained for 10 min.

The fried tilapia meatballs were transferred to a mortar and quickly ground into pieces after a small amount of liquid nitrogen was added. The fragments were transferred into OPP plastic bags and stored in a −80 °C refrigerator (NU-9668E, Nuaire, Plymouth, UK) for the next experiment.

### 2.3. Accelerated Solvent Extraction and High-Vacuum Flavor Extraction Technology (ASE-HVE)

Extractions were carried out using an Accelerated Solvent Extraction Unit (E-914, Buchi, Switzerland). Forty grams of fried tilapia samples, dispersed in 40 g of quartz sand and anhydrous potassium sulfate, was placed into 10 mL inox extraction cells, which were filled with 20 mL dichloromethane and high-purity nitrogen at 55 °C. Then, three static extraction phases that were 8 min each were carried out under 100 Bar [16]. Between extractions, the complete system was rinsed with high-purity nitrogen for 2 min to avoid any carry-over. All extractions were done in triplicate. Extracts were first concentrated using a spiked fractionation column (500 mm × 40 mm) at 48 °C under atmospheric pressure to 150 mL. The extracts were further concentrated under HVE conditions.

The aforementioned extraction liquid from ASE was evaporated by high vacuum (5 × 10^−3^ Pa) distillation using HVE at 50 °C to obtain volatiles. Dichloromethane with the volatiles was slowly separated into a spiked fractionation column (500 mm × 40 mm) at 48 °C under atmospheric pressure to 10 mL volumes and was concentrated to 1 mL under a stream of nitrogen.

### 2.4. Gas Chromatography-Olfactometry (GC-O) and Aroma Extract Dilution Analysis (AEDA)

To identify the aroma compounds in fried tilapia, a gas chromatography-mass spectrometry (GC-MS) instrument (7890A-5977B, Agilent Technologies, Inc., Santa Clara, CA, USA) equipped with an olfactory detection port (ODP3, Gerstel, Germany) was used. A polar HP-INNOWAX capillary column (60 mm × 0.25 mm, 0.25-μm film thickness; J & W Scientific, Folsom, CA, USA) was used, and the column temperature was programmed to increase from 40 °C (after a 3-min hold) to 90 °C at a rate of 10 °C/min (after a 3-min hold), then to 175 °C at a rate of 2 °C/min with a 3-min hold, then to 200 °C at a rate of 5 °C/min with a 3-min hold, and lastly to 240 °C at a rate of 5 °C/min with a 60-min hold. Ultrahigh-purity helium (99.999%, Guangxi Ruida Chemical Technology, Nanning, China) was used as the carrier gas and set at a flow rate of 1 mL/min. An injection volume of 0.5 microliters and electron-impact mass spectra were generated at 70 eV ionization energy with a m/z scan range from 50 to 350. The MS source temperature was 230 °C [17].

AEDA was performed as follows: the concentrated HVE distillate was diluted stepwise with dichloromethane (1:1 by volume), aroma extracts were diluted stepwise with dichloromethane from 1:1 up to 1:6521, and the series of extracts were analyzed by GC-O using a TR-wax column. For each odor-active compound, a flavor dilution (FD) factor was determined, which represented the last dilution in which smell could be perceived. AEDA was performed by 3 trained panelists, and the data obtained were averaged [18].

### 2.5. Gas Chromatography-Mass Spectrometry (GC-MS) Analysis

Volatile compounds from each sample were separated using an Agilent 7890A GC (Agilent Technologies, Palo Alto, CA, USA) equipped with a MS (Agilent Technologies Inc., 5977B VL MSD, Wilmington, DE, USA), an autosampler (Agilent Technologies Inc., 7683 series injectors, Wilmington, DE, USA), and a HP-INNOWAX capillary column (60 mm× 0.25 mm, 0.25-μm film thickness; J & W Scientific, Folsom, CA, USA).

The column temperature was programmed to increase from 40 °C (after a 3-min hold) to 90 °C at a rate of 10 °C/min (after a 3-min hold), to 175 °C at a rate of 2 °C/min with a 3-min hold, to 200 °C at a rate of 5 °C/min with a 3-min hold, and then to 240 °C at a rate of 5 °C/min with a 60-min hold. Ultrahigh-purity helium (99.999%, Guangxi Ruida Chemical Technology, Nanning, China) was used as the carrier gas and set at a flow rate of 1 mL/min. The data acquisition parameters used were within an ion range of 20–300 amu scan mode, detector voltage of 70 eV, and solvent delay of 2 min [18].

The RIs of compounds in the extracts were calculated using the alkane series (C8-C40, 99%, Shanghai, China) that were run using the same GC-MS temperature program for sea urchin roe extracts as described above. The RI of compounds obtained from GC-MS was subsequently used to match the RI obtained from GC-O.

### 2.6. Quantitation of Odor-Active Compounds

The same GC-MS equipment and HP-Innowax chromatography used for qualitative analysis were used for quantitative analysis, and the heating conditions of the column used were consistent with those described above. However, only compounds with significant odor effects were quantified (log_3_FD ≥ 3), and selected ion monitoring was used for quantitative analysis. The fried tilapia concentrates obtained from the ASE-HVE treatment mentioned above were analyzed. Standard curves were established with different concentrations of standard compounds to quantitatively analyze the compounds in the above samples, and three replicates were performed.

The concentration of the odorants in the samples of concentrates was first calculated based on the calibration equation, and the results were the averages of three replicates [19].

### 2.7. Recombination and Omission Experiments

Sensory evaluation was carried out in the sensory room (the room temperature was 23 ± 2 °C), which was followed by ISO 8589 standard [20], with 13 experienced panelists (7 females and 6 males) who were 20–35 years old. The panels were trained with standard solutions and fried tilapia products. After the assessors underwent training, they became familiar with the task and mechanism of the test and the evaluation procedure, and they were determined to be qualified if they passed the tests, which checked their sensory sensitivities in terms of smell [21].

Descriptive vocabularies for recombination experiments ware determined during the training meetings, which involved discussion on sensory attributes of fried tilapia. After comparing with the odor of fried tilapia, the following descriptors were determined to represent the odor characteristics of fried tilapia: grass, roasted, meaty, fishy, and fried-fat.

Sensory evaluations were used for recombination and omission tests [14,22]. An odorless matrix was prepared for the assays. Aroma compounds were extracted five times (8 h each time) from the fried tilapia using a chloroform and methanol mixture (2:1, *v/v*). The meat treated with the chloroform and methanol mixture was dried at 35 °C, distilled water was used, the extraction procedure was repeated, and the fried tilapia were dried under vacuum until the matrix had no smell. The prepared fried tilapia was frozen at −80 °C (NU-9668E, Nuaire, Plymouth, UK). Deodorized matrix (14.5 g) and distilled water (15.5 mL), which was used as the stock recombination solution, were mixed to obtain the recombination model. The water concentration was similar to that found in fried tilapia (51.50 ± 1.02%, which was determined by the thermogravimetric method). The stock recombination solution consisted of a standard reference solution containing 16 compounds with FD values ≥ 27, and its concentration was consistent with that obtained in quantitative analysis in Section 2.6 [9].

The fried tilapia was stored in approximately 25-g portions in thermostat containers before being served warm to sensory booths. Each sample was evaluated in triplicate using the sensory profiling method.

During the odor recombination experiment, the fried tilapia was compared with the recombination model consisting of key aroma compounds, and a 7-point linear scale from 0 (imperceptible) to 3 (strongly feeling) in steps of 0.5 s was evaluated on the flavor profiles of each specified attribute. The experiment was repeated three times, and the results were averaged. Determine the significant differences between the model group and the raw fried tilapia in the above-identified 5 odors by one-way ANOVA.

Triangle tests with forced choices were presented to the assessors in the omission experiment. During the experiment, two samples of the complete recombinant aroma models were evaluated against the omission models (every omission model was recombinant models in which one odorant was omitted). The results of this experiment were evaluated by the binomial distribution [23].

### 2.8. Statistical Analysis

Comparison of the mean values and univariate statistical analysis (one-way ANOVA) were applied to the data obtained in this study using the statistical package SPSS 17.0 (SPSS Inc., Chicago, IL, USA). The significance was established at *p* < 0.05. The aroma recombination experiment data were collected and analyzed by Excel (Microsoft Office 2018, Redmond, WA, USA) [15].

## 3. Results and Discussion

### 3.1. GC-O and AEDA: Aroma-Active Compounds in Fried Tilapia

**Figure 1:**
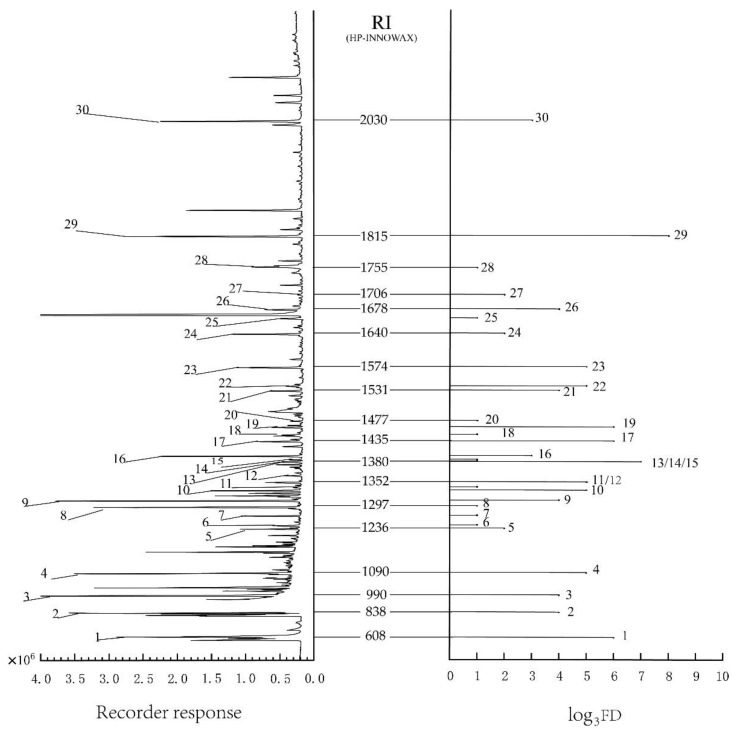
GC-MS and aroma gram of volatile compounds in fried tilapia with FD values and retention indices (RI) on a HP-INNOWAX column. Numbers refer to Table 1.

GC-O analysis and AEDA were used to confirm the potent odorants, which were examined from fried tilapia with the HVE method. The suitable standard considering RI and odor note revealed at the sniffing port was used to confirm each aroma compound. The graphic presentation of volatile compounds with FD values together with GC-MS is shown in Figure 1.

Thirty odorants with FD values ≥ 1 were identified (Table 1). The most intensive of them (FD ≥ 2187) were (E,E)-2,4-decadienal and dimethyl trisulfide, with an odor of fatty meat, and 2,3-dimethyl-5-ethylpyrazine, trimethylamine, and trans-2-octenal, with an FD value of 729 and burnt popcorn, fishy, and cucumber oily smells, respectively. The FD values of 2-methyl-1-propanal, diacetyl, hexanal, acetone alcohol, 2,3-dimethylpyrazine, (E)-2-nonenal, benzaldehyde, and 2-propyl-pyridine were between 81 and 243, with malty, creamy, vegetative, burnt, nutty, cucumber, almond, roasted, and floral aromas. The next compounds had fairly powerful or weaker (3 ≤ FD ≤ 27) fatty, creamy, or candy (1-octen-3-ol, 2-pentyl pyridine, gamma-butyrolactone, (R)-(-)-pantolactone), aldehydic and fruity (octanal, nonanal, 2-pentyl furan, 2-undecenal, (E,E)-2,4-nonadienal), roasted, and earthy or nutty (2-ethyl pyrazine, 2-methyl pyrazine, 2,6-dimethyl pyrazine, 2,3,5,6-tetramethyl pyrazine and 2-ethyl-6-methyl pyrazine) aromas.

The GC-O experiments results show 30 aroma compounds for which the FD factors were greater than or equal to 1. There were 13 heterocyclic compounds containing sulfur or nitrogen, 11 aldehydes (not containing a sulfur atom), 2 lactones, 2 ketones, and 2 alcohols. Among them, 16 compounds had the greatest influence on the fried tilapia since the FD value of the compounds was greater than or equal to 27.

Three furanones identified were found in this study: 2-pentyl furan, furan-2-ylmethanol, and (R)- (-)-pantolactone. These compounds were found in a wide variety of foods, i.e., those that have been detected as key food odorants in more than 25% of the 227 food samples investigated [24]. These are also important intermediates to other flavor compounds, including thiophenes, furan thiols, and other sulfur compounds [25,26,27,28].

Eight pyrazines were prevalent in thermally processed meat. The pyrazines were described in cooked meat of lamb and beef, pork, lamb/mutton, and chicken. 2,3-Dimethyl-5-dimethylpyrazine and 2-ethyl-6-methyl pyrazine are high-impact aromas in roasted and fried meats [29,30]. In contrast, 2-methyl pyrazine, 2,3,5-trimethylpyrazine, and 2,3-dimethylpyrazine were determined to be in Beijing roasted duck although other pyrazines were also found there [31,32].

A total of 10 aldehydes were found in fried tilapia meat. Aldehydes are found in large quantities in fried foods and in the thermal processing products of livestock and poultry. They are considered to be the main odorants of fried foods. Most aldehydes have a low olfactory threshold [33]. (E,E)-2,4-decadienal, trans-2-octenal hexanal, (E)-2-nonenal, and benzaldehyde were found in beef, pork, and bighead carp head soup [34,35], and 2-methyl-1-propanal can be found in cocoa, wine, and cream [36]. Other aldehydes are also found during thermal processing of fatty animal products [37,38].

Two protein degradation products and two thiamine degradation products were found in fried tilapia. Trimethylamine is often used as an important index to measure the freshness of aquatic products [39]; however, as a substance that widely exists in aquatic products, trimethylamine is also an important substance for the characteristic smell of aquatic products, and it is considered to be a key flavor compound of steamed hairy crab and tuna [40,41]. 2-Propylpyridine, as a product of oxidative degradation of proteins, has been reported as an aroma component in fried prawn and peppermint oil [42]. Dimethyl trisulfide has a very low olfactory threshold and has been reported in many meat products and hot, processed aquatic products as one of the sources of meat flavor [43,44]. Recently, there were no relevant reports that 2-hexylthiophene was found in volatile compounds in food, but sensory evaluators could significantly sense the floral odor during GC-O sniffing, which demonstrated that this compound had no significant effect on the odor profile of fried tilapia in the recombination experiment.

Two ketones and three alcohols were found in the volatile aroma compounds of fried tilapia. 2,3-Butanedione was described as having a fatty butter aroma, and it has been discovered as aroma-active compound in several fermented soybean foods. This substance is considered one of the main aroma compounds in soy products and breads [45]. Acetone alcohol is an important intermediate in the formation of pyrazine compounds and has been reported as an important flavor compound in pork soup [46,47]. Amyl alcohol, 1-octen-3-ol, and furan-2-ylmethanol have low olfactory thresholds and have been demonstrated to be key volatile compounds in the aroma studies of fish sauce, brown sugar, pork, Peking duck, etc. [9,43,48,49].

### 3.2. Quantitative Analysis of the Selected Odorants

The sixteen compounds with FD values greater than 27 that were found in AEDA experiments were selected for quantitative analysis. Table 2 summarizes the monitored MS ions, the calibration equations, and the regression coefficient of the standard curve in the quantitative analysis of the GCMS external standard method. The concentration of the odorants in the samples of concentrates was first calculated based on the calibration equation, and the results were the averages of three replicates.

Table 3 summarizes the content of the 16 key detective compounds in tilapia and their calculated olfactory threshold. In terms of quantity, the key volatile compounds of fried tilapia were mainly heterocyclic sulfur- or nitrogen-containing compounds, which accounted for 46.61% of the total compounds and were namely 2-hexylthiophene, 2-propyl-pyridine, 2,3-dimethyl-5-ethylpyrazine, trimethylamine, 2,3,5-trimethyl pyrazine, dimethyl trisulfide, and 2,3-dimethylpyrazine. Many previous studies have shown that sulfur compounds and nitrogen compounds are the main odor compounds of cooked meat products [12,18]. The production of these compounds is often closely related to the mallard reaction of sulfur-containing or nitrogen-containing amino caproic acids or peptides with reducing sugars [50].

It was found aldehydes make up 30.08% of the total compounds and included (E,E)-2,4-decadienal, hexanal, trans-2-octenal, 2-methyl-1-propanal, benzaldehyde, and (E)-2-nonenal. Even though the fat content of tilapia is low, fried oil contains a large number of greases; thus, as a heat transfer medium, oil plays a role in the process of hydrolysis and free fatty acids formation, the further thermal decomposition of saturated fatty acids and unsaturated fatty acids to produce hydrogen peroxide, and the decomposition of hydrogen peroxide to produce large amounts of aldehydes [9].

There were two ketones that made up 12.67% of the total compounds, namely acetone alcohol and diacetyl. In the end, the (R)-(-)-pantolactone one lactone made up 10.64% of the total compound. In the process of frying tilapia, ketones, lactone, and aldehydes were all produced in a similar manner, which was mainly from the oxidation and degradation of fatty acids [51].

### 3.3. Fried Tilapia Aroma Recombination and Deletion Experiment

**Figure 2:**
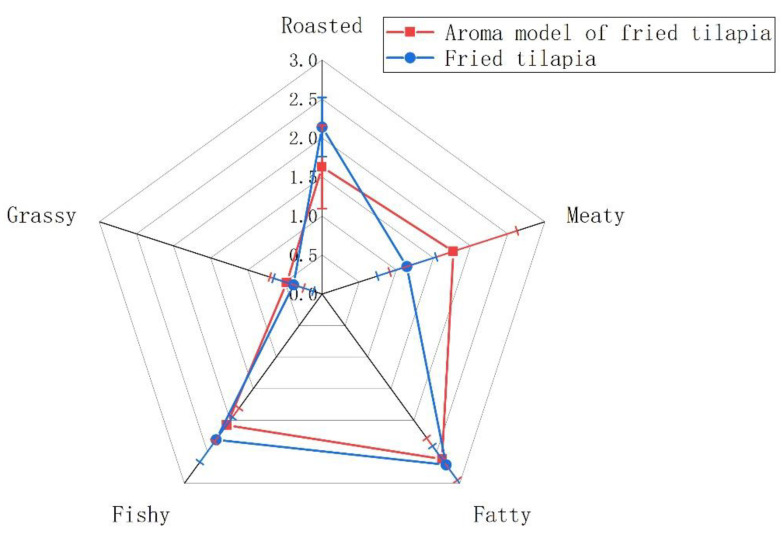
Aroma profiles of fried tilapia compared with the recombination model.

Figure 2 shows that the aroma intensity of each attribute reconstruction model was similar to that of each attribute reconstruction model (*p* > 0.05). These results demonstrate that the selection of odorant active compounds based on AEDA was reasonable, and the qualitative and quantitative experimental results were highly accurate. Therefore, we confirmed that the odor recombination model can reflect the odor characteristics of fried tilapia well.

Omission tests were carried out to further verify the quantitative results. In the 16 potent aroma compounds in the original aroma model mixture, nine key volatiles were found, as shown in Table 4 (*p* < 0.05). These nine volatiles included trimethylamine, hexanal, 2,3-dimethylpyrazine, dimethyl trisulfide, trans-2-octenal, 2,3-dimethyl-5-ethylpyrazine, (E)-2-nonenal, 2-propyl-pyridine, and (E,E)-2,4-decadienal, while other compounds were demonstrated to have no significant contribution to the smell of fried tilapia in the omission experiment (*p* > 0.05).

**Figure 3:**
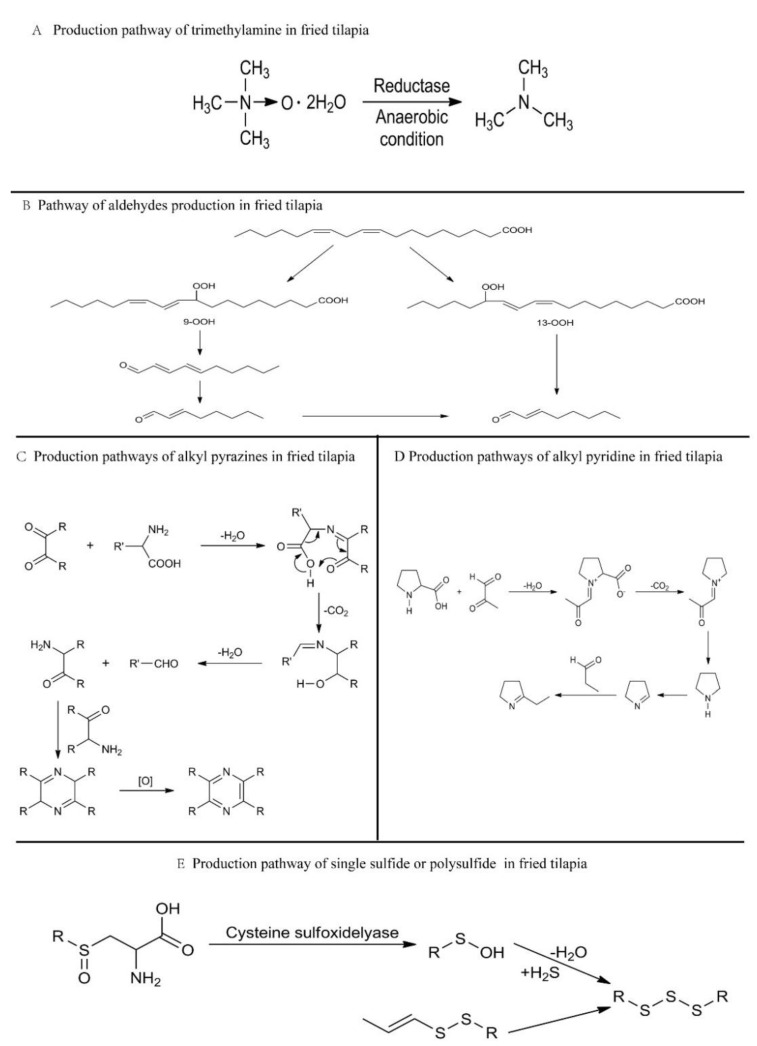
Production pathways of the key aroma compounds in fried tilapia [57,58,59].

The results of this study show that the nine key volatile compounds in fried tilapia are methylamine compounds, aldehyde compounds, heterocyclic compounds, and sulfur compounds. The production mechanism of these compounds in tilapia is extremely complex. Figure 3 summarizes the production mechanism of these compounds in conjunction with the flavor substances contained in tilapia to explore the main sources of volatile compounds.

As a precursor of trimethylamine (TMA), trimethylamine oxide (TMAO) widely exists in aquatic products [52]. In the process of frying tilapia, the oxide is decomposed and produces the main odor substance trimethylamine under the action of enzymes [53], forming the characteristic flavor and slightly fishy odor of fried tilapia. (Figure 3A).

Aldehydes, which are characteristic aromatic substances of fried tilapia, are lipid-derived aldehydes. Although the fat content of tilapia is relatively low (7 ± 0.5%), a large number of fatty acids are involved in the reaction system during the process of frying, resulting in a large number of aldehydes, which become an important source of the characteristic aromas of fried tilapia. For example, the cleavage of 13-hydroperoxide and 9-hydroperoxide results in hexanal and (E,E)-2,4-decadienal, respectively (Figure 3B) [54].

Heterocyclic compounds are mainly generated from the degradation of Strecker aldehydes in the Maillard reaction. These Maillard reactions are mainly generated by the reaction of reducing sugars and amino acids. Tilapia contains a large amount of amino acids and reducing sugars [55,56], which provides a rich substrate for the generation of key pyrazine and pyridine aroma compounds. In the frying process, the oxidation decarboxylation of reducing sugars and amino acids in tilapia produces Strecker aldehydes and α-amrinones, which further condense to form alkane pyrazines, such as 2,3-dimethylpyrazine and 2,3-dimethyl-5-ethylpyrazine, (Figure 3C) to produce key flavor compounds. However, proline and hydroxyproline, which contain the secondary amino group in a pyrrolidine ring, cannot produce α-amrinone and Strecker aldehydes, so they form key flavor substances, such as 2-propyl-pyridine, in the reaction process [57]. (Figure 3D).

Tilapia cysteine produces polysulfides and thiosulfates in the oxidation cracking process during frying, and they further produce dimethyl trisulfide and other single sulfides or polysulfides, resulting in a profuse meat flavor [58] (Figure 3E).

## 4. Conclusions

Applying ASE-HVE-GC-MS/O to fried tilapia resulted in 30 aroma-active components, among which, nine compounds were reported as key characteristic odor compounds of fried tilapia for the first time, to the best of our knowledge. The quantification results indicated that sulfur and nitrogen compounds, aldehydes, ketones, and lipids are the main sources of aroma in fried tilapia. The odor recombination and omission experiments clearly demonstrated that trimethylamine, hexanal, 2,3-dimethylpyrazine, dimethyl trisulfide, trans-2-octenal, 2,3-dimethyl-5-ethylpyrazine, (E)-2-nonenal, 2-propyl-pyridine, and (E,E)-2,4-decadienal were the key volatile compounds in fried tilapia. Finally, the findings not only lay a foundation to further study the flavor components in fried tilapia and flavor regulation during tilapia processing but also provide a theoretical basis for flavor regulation in industrial processing of fried tilapia.

## Figures and Tables

**Table 1:** Aroma-Active Compounds Identified in Fried Tilapia.

NO.	^a^ RI	^b^ Compound	^c^ CAS	^d^ $\log_{3}\;FD$	^e^ Description Odors
1	688	trimethylamine	75-50-3	6	fishy
2	838	2-methyl-1-propanal	78-84-2	4	malty
3	990	diacetyl	431-03-8	4	creamy
4	1090	hexanal	66-25-1	5	vegetative
5	1236	2-pentyl furan	3777-69-3	2	fruity
6	1246	amyl alcohol	71-41-0	1	balsam
7	1274	2-methyl pyrazine	109-08-0	1	nutty
8	1297	octanal	124-13-0	1	orange peel
9	1309	acetone alcohol	116-09-6	4	burnt
10	1334	2,6-dimethyl pyrazine	108-50-9	1	roasted
11	1338	2-ethyl pyrazine	13925-00-3	1	roasted
12	1352	2,3-dimethylpyrazine	5910-89-4	5	nutty
13	1380	dimethyl trisulfide	3658-80-8	7	meaty
14	1391	2-ethyl-6-methyl pyrazine	13925-03-6	1	roasted potato
15	1401	nonanal	124-19-6	1	fatty
16	1409	2,3,5-trimethyl pyrazine	14667-55-1	3	nutty
17	1435	trans-2-octenal	2548-87-0	6	cucumber oily
18	1450	1-octen-3-ol	3391-86-4	1	mushroom
19	1462	2,3-Dimethyl-5-ethylpyrazine	15707-34-3	6	burnt popcorn
20	1477	2,3,5,6-tetramethyl pyrazine	1124-11-4	1	nutty
21	1550	benzaldehyde	100-52-7	4	almond
22	1539	(E)-2-nonenal	18829-56-6	5	cucumber
23	1574	2-propyl-Pyridine	622-39-9	5	roasted
24	1640	gamma-butyrolactone	96-48-0	2	fatty
25	1662	furan-2-ylmethanol	98-00-0	1	bready
26	1678	2-hexylthiophene	18794-77-9	4	floral
27	1706	(E,E)-2,4-nonadienal	5910-87-2	2	fatty
28	1755	2-undecenal	2463-77-6	1	fruity
29	1815	(E,E)-2,4-decadienal	25152-84-5	8	fatty
30	2030	(R)-(-)-pantolactone	599-04-2	3	cotton candy

^a^ Retention index (RI) and odor note at the sniffing port. ^b^ The compounds determined in the quantitative analysis. ^c^ The unique numerical identifier assigned by the Chemical Abstracts Service (CAS). ^d^ Odors perceived at the sniffing port. ^e^ Flavor dilution factor on the HP-INNOWAX column.

**Table 2:** Authentic Chemicals, Scanned Ions, Calibration Equations, and Recovery Factors in the Quantitative Determination of Odorants by HVE Combined with GC-MS in a Selected Ion Monitoring (SIM) Mode.

^a^ RI	^b^ Name	^c^ Ionsd (m/z)	^d^ Calibration Eqs	^e^ R^2^
866	trimethylamine	58, 59, 42	y = (x − 130,147.5252) ÷ 34,398.9676x	0.9925
955	2-methyl-1-propanal	43, 41, 72	y = (x + 19,055.1351) ÷ 81,569.0453x	0.9958
1014	diacetyl	43, 86, 42	y = (x + 25,952.8011) ÷ 78,372.0369x	0.9985
1091	hexanal	44, 56, 41	y = (x + 64,514.4616) ÷ 16,148.8895x	0.9931
1309	acetone alcohol	43, 31, 74	y = (x + 117,988.5619) ÷ 89,266.6282x	0.9977
1349	2,3-dimethylpyrazine	67, 108, 40	y = (x + 19,338.4727) ÷ 79,528.563618x	0.9975
1394	dimethyl trisulfide	126, 45, 79	y = (x + 91,851.5539) ÷ 90,629.4553x	0.9983
1406	2,3,5-trimethyl pyrazine	42, 122, 39	y = (x + 25,713.2766) ÷ 82,365.0994x	0.9968
1435	trans-2-octenal	41, 55, 29	y = (x + 15,351.2721) ÷ 14,161.1804x	0.9963
1462	2,3-Dimethyl-5-ethylpyrazine	135, 136, 108	y = (x + 2351.1146) ÷ 9868.1848x	0.9962
1531	benzaldehyde	77, 106, 105	y = (x + 49,893.6100) ÷ 57,871.0904x	0.9971
1539	(E)-2-nonenal	43, 55, 70	y = (x + 26,133.6678) ÷ 23,108.7456x	0.9969
1574	Pyridine, 2-propyl-	93, 106, 120	y = (x + 25,621.8714) ÷ 20,115.1258x	0.9961
1678	2-hexylthiophene	97, 98, 168	y = (x + 15,988.1955) ÷ 13,139.4778x	0.9959
1811	(E,E)-2,4-decadienal	81, 41, 39	y = (x − 607,760.6942) ÷ 82,363.2326x	0.9914
2030	(R)-(-)-pantolactone	71, 43, 41	y = (x + 233,527.6726) ÷ 48,525.7271x	0.9950

^a^ Retention indices on the HP-INNOWAX (60 m × 0.25 mm × 0.25 μm) column in GC-MS analysis. ^b^ The compounds determined in the quantitative analysis. ^c^ The monitored ions used in the quantitation. ^d^ Standard curve regression equation established by the external standard method. ^e^ Regression coefficient of the standard curve in quantitative analysis of the GC-MS external standard method.

**Table 3:** Odor Thresholds by GC-O, Odor Thresholds in Air, Analyte Concentration in the Sample Solution, and Odor Activity Values of the Aroma-Active Compounds of Fried Tilapia.

RI	Name	^a^ FD	CAS	^b^ Concentration (mg/kg)	^c^ Olfactory Threshold (mg/kg)
866	trimethylamine	729	75-50-3	78.6441 ± 9.4903	0.1079 ± 0.0228
955	2-methyl-1-propanal	81	78-84-2	10.9421 ± 1.8490	0.1351 ± 0.0228
1014	diacetyl	81	431-03-8	16.6671 ± 2.7772	0.2058 ± 0.0343
1091	hexanal	243	66-25-1	78.6330 ± 3.1470	0.3236 ± 0.0130
1309	acetone alcohol	81	116-09-6	95.4288 ± 5.6692	1.1781 ± 0.0278
1349	2,3-dimethylpyrazine	243	5910-89-4	3.9575 ± 0.7406	0.0163 ± 0.0030
1394	dimethyl trisulfide	2187	3658-80-8	5.3529 ± 0.5088	0.0024 ± 0.0002
1406	2,3,5-trimethyl pyrazine	27	14667-55-1	41.2071 ± 3.3091	1.5262 ± 0.1226
1435	trans-2-octenal	729	2548-87-0	42.1350 ± 4.0492	0.0578 ± 0.0056
1462	2,3-Dimethyl-5-ethylpyrazine	729	15707-34-3	89.9714 ± 3.8457	0.1234 ± 0.0053
1531	benzaldehyde	81	100-52-7	9.5011 ± 0.6848	0.1173 ± 0.0085
1539	(E)-2-nonenal	243	18829-56-6	4.5697 ± 0.2256	0.0188 ± 0.0009
1574	Pyridine, 2-propyl-	243	2294-76-0	93.4565 ± 9.3617	0.3846 ± 0.0263
1678	2-hexylthiophene	81	18794-77-9	99.9169 ± 7.9401	1.2335 ± 0.1645
1811	(E,E)-2,4-decadienal	6561	25152-84-5	120.3953 ± 1.5619	0.0184 ± 0.0002
2030	(R)-(-)-pantolactone	27	599-04-2	94.1932 ± 8.8211	3.4886 ± 0.3267

^a^ Flavor dilution (FD) factor determined by a HP-INNOWAX column. ^b^ The concentration of key volatile flavor compounds in fried tilapia was quantitatively analyzed by GC-MS. ^c^ The odor activity value was calculated by dividing the concentration of the analyte in the sample solution (mg/mL) by the dilution ratio obtained by sniffing through the olfactory mouth.

**Table 4:** Results of the omission experiments for the fried tilapia model.

No.	Compound(s) Omitted	Significance
1	trimethylamine	***
2	2-methyl-1-propanal	NS
3	diacetyl	NS
4	hexanal	*
5	acetone alcohol	NS
6	2,3-dimethylpyrazine	**
7	dimethyl trisulfide	***
8	2,3,5-trimethyl pyrazine	NS
9	trans-2-octenal	*
10	2,3-Dimethyl-5-ethylpyrazine	**
11	benzaldehyde	NS
12	(E)-2-nonenal	*
13	2-propyl-pyridine	**
14	2-hexylthiophene	*
15	(E,E)-2,4-decadienal	***
16	(R)-(-)-pantolactone	NS

NS, no significant difference; *** 0.01% significance level; ** 0.1% significance level; * 0.5% significant level.

## Data Availability

The data showed in this study are contained within the article.
